# Disparities Made Invisible: Gaps in COVID-19 Data for American Indian and Alaska Native Populations

**DOI:** 10.1089/heq.2021.0129

**Published:** 2022-03-08

**Authors:** Alexandra Skinner, Julia Raifman, Elizabeth Ferrara, Will Raderman, Talia M. Quandelacy

**Affiliations:** ^1^ Department of Health Law, Policy and Management, Boston University School of Public Health, Boston, Massachusetts, USA.; ^2^ Department of Epidemiology, Colorado School of Public Health, Aurora, Colorado, USA.

**Keywords:** COVID-19, American Indian/Alaska Native, data collection, data reporting, health disparities

## Abstract

**Introduction:**

Complete COVID-19 data for American Indian/Alaska Native (AI/AN) populations are critical to equitable pandemic response.

**Methods:**

We used the COVID-19 U.S. State Policy database to document gaps in COVID-19 data reporting for AI/AN people.

**Results:**

Sixty-four percent of states do not report AI/AN data for at least one COVID-19 health metric: cases, hospitalizations, deaths, or vaccinations.

**Discussion:**

The lack of AI/AN-specific data masks the disproportionate burden of COVID-19 and presents challenges to COVID-19 prevention, policy implementation, and health equity.

**Conclusions:**

Public-facing data disaggregated by race may facilitate rapid response COVID-19 research and policymaking to support AI/AN communities.

## Introduction

Data on COVID-19 and other health outcomes by race and ethnicity are essential for targeting resources and evaluating policies and programs to reach those with the greatest need. In particular, the COVID-19 pandemic illuminates systemic underreporting and misclassification by race of American Indian/Alaska Native (AI/AN) people in vital records. A lack of distinguishable AI/AN data perpetuates racial inequities in COVID-19 outcomes and in the allocation of COVID-19 resources for treatment and prevention.

Historical genocide, land theft, and the exploitation of Indigenous resources and culture shape inequities in wealth, chronic disease, and life expectancy for AI/AN individuals.^1^ In the context of the pandemic, limited data indicate that AI/AN people between the ages of 25 and 64 years are 4.3–19.1 times as likely to die of COVID-19 relative to non-Hispanic white people.^2^ Yet prepandemic misclassification of AI/AN deaths has been reported as up to 40%. Thus, there is reason for significant concern over the accuracy of COVID-19 AI/AN data, as true health disparities figures are likely even larger than reported.^3^

Other health and economic indicators are not reported at all for AI/AN populations, even if data may be available. The National Center for Health Statistics released preliminary life expectancy estimates for 2020 and found that life expectancy dropped substantially for all racial groups, and more so for those that are non-white.^4^ However, AI/AN people are excluded from this report.

Similarly, the U.S. Department of Agriculture measured household food security in the United States before the pandemic, but did not report food security status for AI/AN households.^5^ As a result, research questions that investigate how the pandemic has impacted food insecurity among AI/AN people remain unanswered. These incomplete data present substantial challenges to public health fieldwork, policy implementation, and health equity.

Prior research confirms that health and mortality assessments for AI/AN populations are often hindered by a lack of complete and accurate data on race and ethnicity in surveillance systems.^6^ However, the extent to which COVID-19 research and policymaking are limited by missing or misclassified AI/AN data is not well understood. Yellow Horse and Huyser suggest that reliable COVID-19 data at the state level will be critical in eliminating the disproportionate impact of COVID-19 on AI/AN peoples.^7^ This article aims to identify states that do not publicly release AI/AN-specific COVID-19 case, hospitalization, mortality, and vaccination data to highlight the gaps in our understanding of the pandemic's impact on AI/AN communities.

## Methods

To document state COVID-19 data reporting for AI/AN populations in December 2021, we used the COVID-19 U.S. State Policy (CUSP) database, a collection of state orders in response to the pandemic and its economic ramifications.^8^ CUSP documents whether state health departments publicly reported COVID-19 cases, hospitalizations, deaths, and vaccinations by race and ethnicity, and indicates whether AI/AN is included as a distinct race category. Although states may release additional racial data through the National Center for Health Statistics, this data source notes a time lag and is more likely to suppress data for non-white populations because these populations are typically smaller in size.^9^

## Results

COVID-19 cases are reported for AI/AN populations more widely than are COVID-19 hospitalizations, deaths, and vaccinations. Yet even for COVID-19 cases, AI/AN people are underrepresented in state data reporting. From June 30 to September 20, 2021, the Nebraska Department of Health and Human Services removed its COVID-19 data dashboard from its website. As a result, COVID-19 data on any outcomes by race and ethnicity were not publicly accessible through the state of Nebraska for several months during a nationwide COVID-19 surge due to the delta variant. In addition, as of December 2021, New York does not report COVID-19 cases by race or ethnicity and North Dakota and West Virginia have not publicly released race-specific COVID-19 mortality data.

**FIG. 1.**
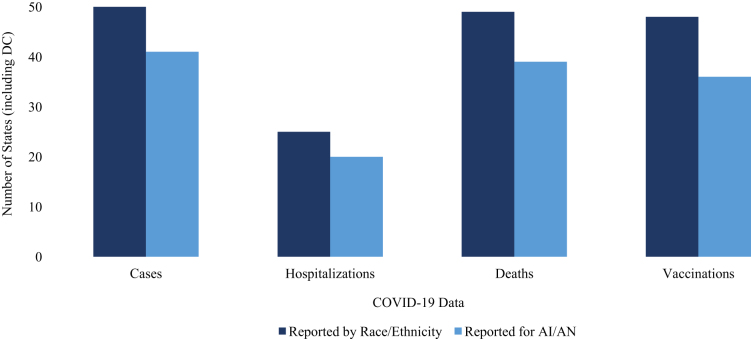
States reporting race-specific COVID-19 health metrics. This figure depicts the number of states that report COVID-19 cases, hospitalizations, deaths, and vaccinations by race and ethnicity and for AI/AN populations specifically. AI/AN, American Indian/Alaska Native.

An additional eight states fail to report case and mortality figures for AI/AN people (Fig. 1). Although 50% of states report COVID-19 hospitalization data by race and ethnicity, just 40% explicitly report AI/AN hospitalizations. COVID-19 vaccination data by race and ethnicity is publicly available for 96% of states and the District of Columbia, but only 72% report data specifically for AI/AN people. Several states do not report AI/AN data across any COVID-19 health metrics, including Texas and New York—homes to the country's third and fifth largest AI/AN populations, respectively.^10^ In fact, only five of the states with the 10 largest AI/AN populations report COVID-19 cases, hospitalizations, deaths, and vaccinations for AI/AN people.

## Discussion

CUSP data indicate that 64% of U.S. states and the District of Columbia do not report all COVID-19 data for AI/AN people. The Urban Indian Health Institute (UIHI) similarly finds that more than half of states scored grades of “C” or lower on an assessment of the quality of state-reported COVID-19 racial data on AI/AN populations.^10^ Many state health departments omit AI/AN identities by combining their data in an “other” category, contrary to UIHI guidelines to define AI/AN populations as AI/AN alone. This practice masks the disproportionate burden of COVID-19 in AI/AN communities and renders AI/AN people invisible amidst state, federal, and tribal policy discussions surrounding resource allocation.

COVID-19 case data from tribes are internally reported to the Indian Health Service and provided to state and federal agencies; however, case data from urban AI/AN populations—that make up ∼70% of AI/AN people in the United States^11^—are limited and add challenges in understanding the full impact of COVID-19. Furthermore, health data are rarely disaggregated by tribal affiliation or tribal enrollment status, which limits the extent to which states can identify disparities among AI/AN subpopulations.^12^

Aggregating data by race also diminishes the strengths of the AI/AN approach to COVID-19 mitigation. AI/AN communities initially had the highest rates of vaccination of all racial and ethnic groups,^13^ but without easily accessible public-facing data from states and the Indian Health Service, discourse about vaccine and booster distribution strategies overlooks the success of AI/AN people. At the same time, new and more transmissible variants drive surges in COVID-19 cases among AI/AN populations,^14^ but data on breakthrough infections are typically not disaggregated by race and ethnicity. Complete data would allow researchers and policymakers to better understand transmission of COVID-19 variants even among groups with high vaccination rates.

COVID-19 reporting is not standardized and there may be more granular data available to those working in state or federal health departments. These data may not be captured by publicly available data sets. Even so, disaggregated data by race and ethnicity should be made available to all relevant stakeholders, including policymakers, researchers, and the media. Our analysis is additionally limited by its cross-sectional design as our data do not reflect policy changes over time.

## Conclusions

Complete COVID-19 data by race and ethnicity are necessary to inform continued COVID-19 prevention measures as well as subsequent policy interventions and vaccination campaigns. The surge of the delta variant and record-breaking transmission of the omicron variant presented additional challenges to COVID-19 mitigation, yet state surveillance systems continue to overlook the impact on AI/AN communities. As the COVID-19 pandemic lays bare historical and modern-day policies that have shaped a highly inequitable world, standardization of AI/AN data for all health and economic indicators can inform a more equitable future.

## Abbreviations Used

AI/AN: American Indian/Alaska Native
CUSP: COVID-19 U.S. State Policy
UIHI: Urban Indian Health Institute
